# Pyruvate Kinase M2 Is Required for the Expression of the Immune Checkpoint PD-L1 in Immune Cells and Tumors

**DOI:** 10.3389/fimmu.2017.01300

**Published:** 2017-10-13

**Authors:** Eva M. Palsson-McDermott, Lydia Dyck, Zbigniew Zasłona, Deepthi Menon, Anne F. McGettrick, Kingston H. G. Mills, Luke A. O’Neill

**Affiliations:** ^1^School of Biochemistry and Immunology, Trinity College Dublin, Trinity Biomedical Science Institute, Dublin, Ireland

**Keywords:** PD-L1, pyruvate kinase isoform M2, LPS, macrophages, dendritic cells, tumor cells, hypoxia-inducible factor 1α

## Abstract

Blocking interaction of the immune checkpoint receptor PD-1 with its ligand PD-L1 is associated with good clinical outcomes in a broad variety of malignancies. High levels of PD-L1 promote tumor growth by restraining CD8^+^ T-cell responses against tumors. Limiting PD-L1 expression and function is therefore critical for allowing the development of antitumor immune responses and effective tumor clearance. Pyruvate kinase isoform M2 (PKM2) is also a key player in regulating cancer as well as immune responses. PKM2 catalyzes the final rate-limiting step of glycolysis. Furthermore, PKM2 as a dimer translocates to the nucleus, where it stimulates hypoxia-inducible factor 1α (Hif-1α) transactivation domain function and recruitment of p300 to the hypoxia response elements (HRE) of Hif-1α target genes. Here, we provide the first evidence of a role for PKM2 in regulating the expression of PD-L1 on macrophages, dendritic cells (DCs), T cells, and tumor cells. LPS-induced expression of PD-L1 in primary macrophages was inhibited by the PKM2 targeting compound TEPP-46. Furthermore, RNA silencing of PKM2 inhibited LPS-induced PD-L1 expression. This regulation occurs through direct binding of PKM2 and Hif-1α to HRE sites on the PD-L1 promoter. Moreover, TEPP-46 inhibited expression of PD-L1 on macrophages, DCs, and T cells as well as tumor cells in a mouse CT26 cancer model. These findings broaden our understanding of how PKM2 may contribute to tumor progression and may explain the upregulation of PD-L1 in the tumor microenvironment.

## Introduction

Immune checkpoint interactions play a crucial role in host maintenance of immune homeostasis. These include CTLA-4:CD80/CD86, PD1:PD-L1/PD-L2, GAL9:TIM3, TCR:LAG3, and HVEM:BTLA, all of which can contribute to the suppression of T cell effector function in the tumor microenvironment. This suppression supports tumor progression, a process that has been termed “immunoediting,” and which has been the focus of extensive efforts to restore host antitumor immunity during immunotherapy.

PD-L1 is a key player in establishing an immunoevasive tumor milieu (1–3). Evidence from preclinical and clinical trials suggests that antibody-mediated disruption of the PD-1 and PD-L1 complex enhances antitumor immunity, with several approved antibodies targeting this interaction now in use (4). PD-L1 is a member of the B7 family of costimulatory/coinhibitory molecules and is expressed on a wide range of cell types, including cancer cells (3, 5). PD-L1 expression is regulated by interferon regulatory factor-1 and signal transducer and activator of transcription 1 (6). In addition, PD-L1 upregulation during hypoxia requires hypoxia-inducible factor 1α (Hif-1α) (7). PD-L1 expression correlates with disease progression in several solid cancers, such as ovarian and pancreatic cancer (8, 9), as well as renal cell carcinoma (10). Therefore, a clear understanding of how expression of PD-L1 is regulated is critical in the design of novel combination cancer immunotherapies.

Pyruvate kinase isoform M2 (PKM2) has also been linked with tumor progression. Pyruvate kinase is the rate-limiting enzyme that converts phosphoenolpyruvic acid (PEP) to pyruvate. In resting immune cells and differentiated tissue, this reaction is mediated by PKM1. However, during the metabolic reprogramming, which takes place in activated immune cells and in tumor cells, expression of PKM1 decreases in favor of PKM2. PKM2 exists primarily as an enzymatically inactive monomer or dimer, a stage when PKM2 can translocate to the nucleus. Here, in a complex with Hif-1α and p300, PKM2 acts as a coactivator of Hif-1α, a process that is dependent on prolyl hydroxylase 3 (PHD3), thereby regulating expression of numerous proglycolytic enzymes and further contributing to tumorigenesis (11).

Here, we provide the first direct link between two of the central players in cancer, PKM2 and PD-L1, providing new mechanistic insight into the regulation of PD-L1 expression by PKM2 on tumor cells, but more importantly on a range of immune cells. Our findings may provide novel therapeutic direction in the growing field of combination immunotherapy for cancer.

## Materials and Methods

### Reagents

TEPP-46 and DASA-58 were synthesized in accordance with published methods (12, 13) and were a kind gift from Craig Thomas, NIH, MD, USA. LPS used was *E. coli*, serotype EH100 (Alexis). Western blotting antibodies were anti-PKM2 D78A4 (4053) and anti-β-actin (4267) from Cell Signaling Technologies, anti-mB7-H1 (PD-L1, AF1019) from R&D Systems, and anti-HIF-1α (NB100-449) from Novus. Anti-mouse-CD274PE (MIH5) from eBioscience and anti-mouse CD16/CD32 (Mouse BD Fc Block) from BD Pharmingen™ were used for FLOW cytometry *in vitro*. SYBR primers were from Eurofins, UK. Dimethyl sulfoxide used as vehicle control for TEPP-46 in all relevant *in vitro* assays. Antibodies used for flow cytometry analysis of *in vivo* tissues are listed in Table S1 in Supplementary Material.

### Mice and Cell Culture

Bone marrow-derived macrophages and bone marrow-derived dendritic cells (BMDCs) were isolated from C57BL/6 mice (Envigo UK, female; 8–12 weeks old, maintained under specific pathogen-free conditions in line with Irish and European Union regulations) as previously described (14). All experiments were carried out with prior ethical approval from Trinity College Dublin Animal Research Ethics Committee. Cells were used at 1 × 10^6^ cells/ml unless otherwise stated. Each “*n*” represents BMDM/BMDC from one individual mouse. The RAW264.7 (mouse leukemic monocyte macrophage) cell line was obtained from Sigma-Aldrich.

### Flow Cytometry Measuring PD-L1 in BMDM Cells

Bone marrow-derived macrophage cells were seeded at 1 × 10^6^ cells in 1 ml in a 24-well plate. While on ice cells were washed three times in Flow Buffer (PBS, 0.5% fetal bovine serum, 2 mM EDTA) and incubated with Fc Block (1:100) and CD274PE (1:50) in 100 µl Flow Buffer for 15 min, 4°C in dark. After three washes in Flow Buffer, PD-L1 surface expression was measured by Flow Cytometry.

### RNA and Gene Expression

Total RNA was isolated using PureLink™ RNA Mini Kit (Ambion by Life Technologies). RNA was transcribed using High-Capacity cDNA Reverse Transcription Kit (Applied Biosystems). For mRNA, 18S ribosomal RNA, GAPDH, or RPS18 gene were used as housekeeping controls. SYBR probes were from Eurofins, UK (sequences as shown in Table S2 in Supplementary Material). Relative quantitation values were calculated using the 2(−Delta Delta *C*(*T*)) method (15). All fold changes are expressed normalized to untreated control.

### siRNA Transfection

Bone marrow-derived macrophage cells were plated at 1 × 10^6^ cells/ml in a 24-well plate. Transfections were performed in serum and antibiotic-free media supplemented with 10% L929 media. Transfections using Lipofectamine RNAiMAX (13778-150) from Invitrogen were carried out according to the manufacturer’s recommendations using a final concentration of 10 nM Pkm2 Silencer^®^ Select Pre-designed siRNA (4390771) or scrambled control (SC) siRNA from Ambion. Western blot analysis was carried out as previously described (16) and bands were visualized using Gel Doc™ EZ gel imaging system.

### Oligonucleotide Pull Down Assay

Oligonucleotides for the HRE sites on the PD-L1 were annealed as previously described (17) (sequences as shown in Table S3 in Supplementary Material).

Bone marrow-derived macrophages (1 × 10^6^ cell/ml in 10 ml) were treated with TEPP-46 (50 µM) for 1 h prior to LPS (24 h, 100 ng/ml). Oligonucleotide pull down was performed as previously described (17). Western blot analysis was carried out as previously described (16) and bands were visualized using Gel Doc™ EZ gel imaging system.

### Chromatin Immunoprecipitation (ChIP)

The antibodies used were rabbit anti-PKM2 D78A4 (Cat. 4053, Cell Signaling Technology), rabbit Hif-1α (ab2185, Abcam), and rabbit IgG (PP64, Millipore). For regular ChIP, the immunoprecipitated DNA was quantified by real-time qPCR. For sequential ChIP, eluted DNA from the initial immunoprecipitation using anti-Hif-1α was incubated with beads to remove unbound rabbit anti-Hif-1α prior to incubation with anti-PKM2 as described previously (18). Sequences of the PCR primers were as previously described (7). All ChIP and Re-ChIP analyses were done by ddCT method and values were corrected for non-specific binding of respective IgG control and thereafter represented as percentage input (18). For sequential ChIP, Hif-1α-bound chromatin resulting from first ChIP step was used as input value.

### Tumor Model

The CT26 murine colon carcinoma cell line was purchased from the ATCC (Manassas, VA, USA). BALB/c mice were injected s.c. into the flank with 3 × 10^5^ CT26 tumor cells. TEPP-46 (50 mg/kg) or vehicle control (30% cyclodextrin) were injected i.p. every 2–3 days starting on day −1 before tumor induction. On day 15 post-tumor induction, mice were sacrificed and tumors and tumor draining lymph nodes (dLN) dissected. Tumors were chopped and digested with DNAse I (20 U/ml) and collagenase D (1 mg/ml) for 1 h. Single cell suspensions were prepared using a 100-µm nylon mesh and stained for flow cytometry or prepared for RT-PCR.

### Flow Cytometry of *In Vivo* Tissue Samples

Cells were stained with FACS antibodies (Table S1 in Supplementary Material). Data were acquired using LSR Fortessa™ (BD) and analyzed using FlowJo software. The gating strategy is shown in Figure S1 in Supplementary Material.

### Statistical Analysis

Comparisons between two groups were calculated using two-tailed Student’s *t*-tests, using GraphPad Prism software. Data are reported as mean ± SEM. Statistical values, including number of replicates (*n*), can be found in the figure legends. *P < 0.05, **P < 0.01, ***P < 0.001. For *in vitro* experiments, *n* = number of separate experiments. For *in vivo* work, *n* = number of individual animals.

## Results

### PKM2 Expression and Function Play a Key Role in LPS-Induced PD-L1 Expression of Activated Macrophages and Dendritic Cells (DCs)

**Figure 1:**
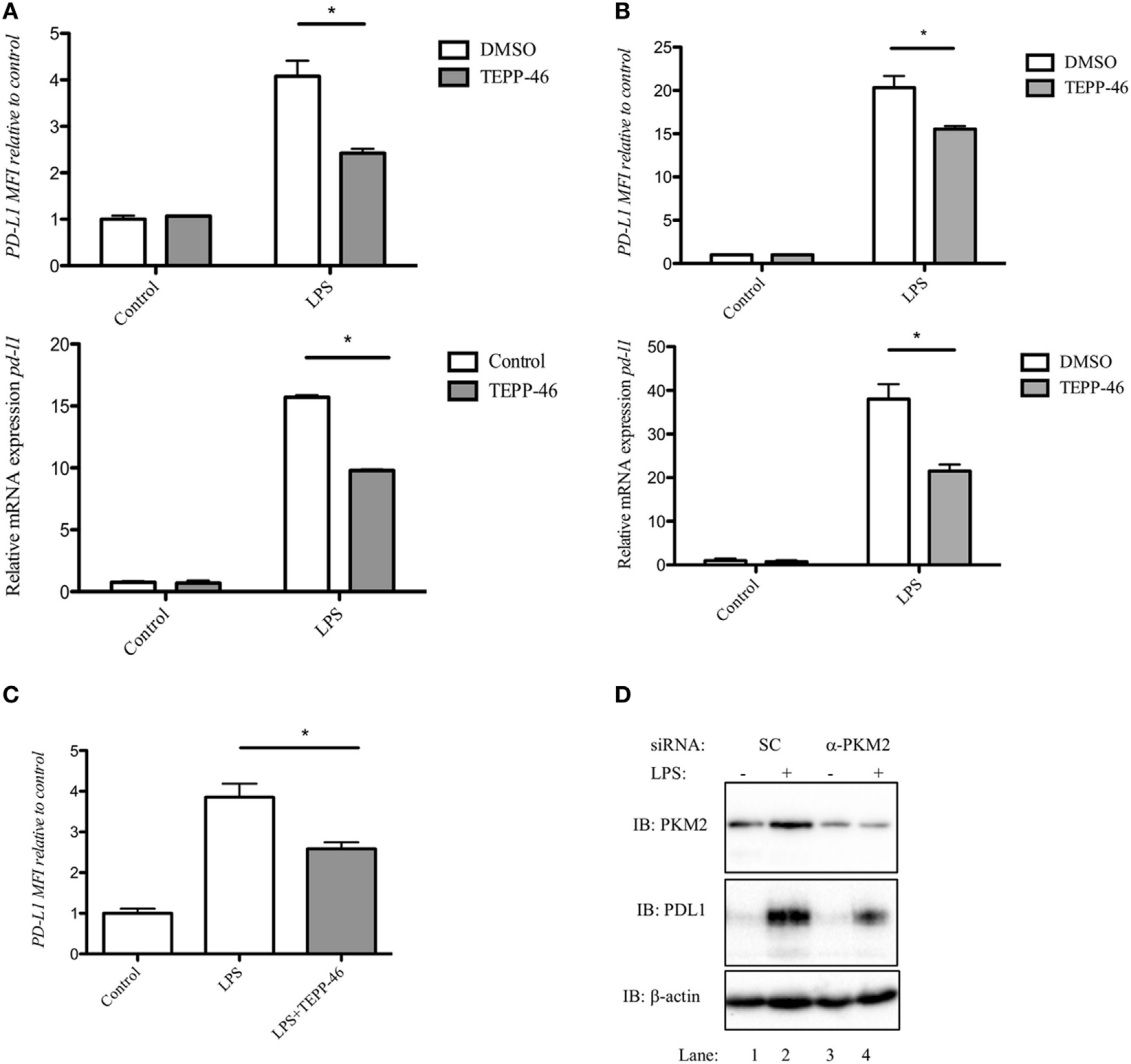
Pyruvate kinase isoform M2 (PKM2) is required for LPS-induced expression of PD-L1. RAW cells (0.5 × 10^6^ cells/ml) **(A)**, bone marrow-derived macrophages (BMDMs) **(B)**, or bone marrow-derived dendritic cells **(C)** were treated with TEPP-46 at 50 µM for 1 h prior to stimulation with LPS (100 ng/ml, 24 h), lysed and assayed for expression of PD-L1 by flow cytometry [**(A–C)** top panels] and *pdl1* mRNA by RT-PCR [**(A,B)** bottom panels]. Statistical analysis was performed on cells from three separate experiments. Error bars represent mean ± SEM. BMDM cells were transfected with scrambled control (SC) or anti-PKM2 siRNA **(D)**. 24 h post-transfection, cells were stimulated with LPS (100 ng/ml) for 24 h after which expression of PKM2 (top panel) and PDL1 (middle panel) was measured by western blot.

Since PD-L1 is important in maintaining immune homeostasis by regulating an overactive immune response, we first examined expression of PD-L1 in activated macrophages and DCs. Stimulation of RAW 264.7 macrophages (Figure 1A), murine bone marrow-derived macrophages (BMDM, Figure 1B), or murine BMDC (Figure 1C) with LPS for 24 h (100 ng/ml), induced a robust increase in expression of PD-L1 surface protein (top panels), and *pdl1* RNA transcript (bottom panels).

PD-L1 was recently shown to be regulated by Hif-1α during hypoxia. We next investigated whether modifying PKM2 using the small molecule activator TEPP-46 would alter the expression of LPS-induced PD-L1 in immune cells. Treatment of RAW 264.7 cells with 50 µM TEPP-46 prior to stimulation with LPS for 24 h significantly reduced surface expression of PD-L1 protein and *pdl1* mRNA transcript (Figure 1A). Furthermore, TEPP-46 inhibited LPS-induced expression of PD-L1 protein (top panel) and *pdl1* mRNA (bottom panel) in murine macrophages (Figure 1B) and DCs (Figure 1C), suggesting a role for PKM2 in regulating PD-L1 expression.

To further demonstrate the importance of PKM2 in LPS-induced PD-L1 expression, BMDM cells were transfected with silencing RNA targeting PKM2. siRNA targeting PKM2 significantly reduced expression of PKM2 protein compared to SC (Figure 1D; top panel, compare lanes 3 and 4 to lanes 1 and 2). As a consequence of reduced PKM2 expression, LPS-induced PD-L1 expression was decreased (Figure 1D, middle panel, compare lane 3 and 4, α-PKM2, to lanes 1 and 2, SC) further implicating a role for PKM2 in PD-L1 expression in macrophages.

### PKM2 and Hif-1α Bind Directly and Simultaneously to Two HRE Promoter Regions of the PD-L1 Promoter

**Figure 2:**
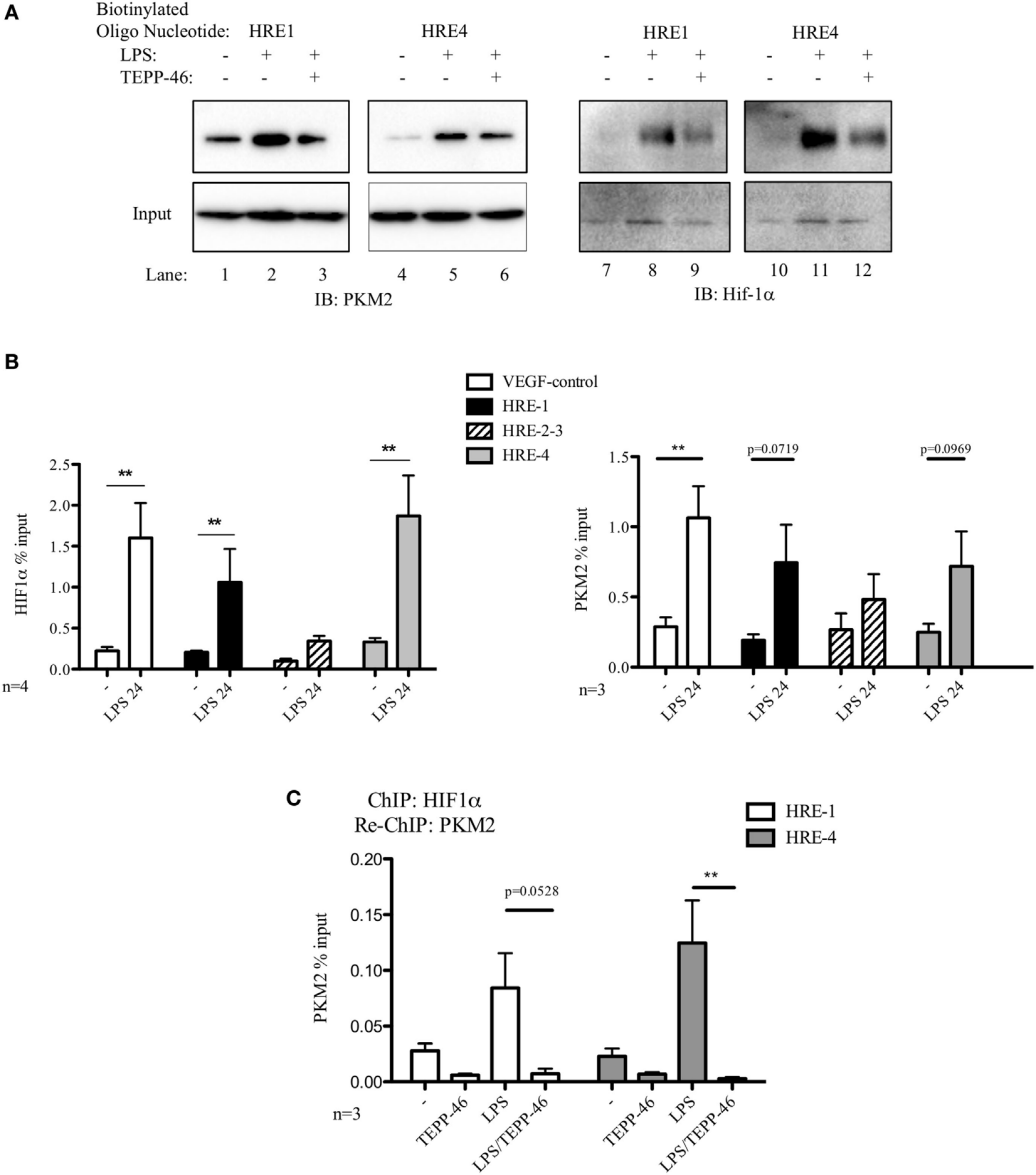
Pyruvate kinase isoform M2 (PKM2) and hypoxia-inducible factor 1α (Hif-1α) bind directly to the promoter region of PDL1 in primary murine BMDM cells. BMDM cells were treated with TEPP-46 at 50 µM for 1 h prior to stimulation with LPS (100 ng/ml, 24 h) **(A)**. Binding of PKM2 (left) or Hif-1α (right) to HRE1 or HRE4 of the PD-L1 promoter was detected by incubating cell lysates with biotinylated oligonucleotides spanning the relevant HRE promoter region. Protein–oligonucleotide complexes were isolated using streptavidin agarose beads, and proteins were detected by western blotting. Representative of *n* = 3. Chromatin immunoprecipitation (ChIP)-PCR **(B)** using PKM2 and HIF-1α antibodies and primers specific for three promoter regions of PD-L1 (HRE1, 2–3, and 4) and a known Hif-1α binding region of vascular endothelial growth factor as a positive control showing binding of Hif-1α (left) and PKM2 (right) to the PD-L1 promoter in LPS-treated BMDMs (100 ng/ml, 24 h). **(C)** Sequential ChIP assays measuring simultaneous endogenous binding of PKM2 and Hif-1α to chromatin in response to LPS (100 ng/ml, 24 h) ±TEPP-46 (pretreatment using 50 µM, 60 min). ChIP data are calculated as percent of input, error bar represents mean ± SEM, and statistics are performed as two-tailed unpaired *t*-test *P < 0.05, **P < 0.01, and ***P < 0.001.

We next designed biotinylated oligonucleotides spanning two of the hypoxia response elements (HRE) present in the PD-L1 promoter, HRE1 and HRE4, which are known to bind Hif-1α during hypoxia (7). As seen in Figure 2A, endogenous PKM2 (left panels) as well as Hif-1α (right panels) bind to the HRE1 and HRE4 elements of the PD-L1 promoter in primary macrophages. PKM2 binds basally to the promoter in resting BMDM cells (2A, lanes 1 and 4 top panel), however, binding of Hif-1α and of PKM2 to the two oligonucleotide sequences are significantly increased in response to LPS (compare lanes 1–2, 4–5, 7–8, and 10–11, top panel). Furthermore, modifying PKM2 into a tetrameric configuration using TEPP-46 reduces binding of both PKM2 itself as well as Hif-1α to the promoter sequences (Figure 2A, top panels, compare lanes 2–3, 5–6, 8–9, and 11–12).

To definitively confirm the observed binding of PKM2 and Hif-1α to the PD-L1 promoter, we next performed ChIP assays on lysates from primary murine BMDM cells. Chromatin-bound endogenous Hif-1α (Figure 2B, left) and PKM2 (Figure 2B, right) complexes were isolated using relevant antibodies as indicated, and bound DNA was identified using Q-PCR. Assay integrity was verified using a known Hif-1α-binding site of the vascular endothelial growth factor (VEGF) promoter region. LPS stimulation enhanced binding of endogenous Hif-1α to the VEGF promoter in primary BMDM cells (Figure 2B, left). Noman et al. reported that Hif-1α regulates PD-L1 expression by directly binding to the PD-L1 proximal promoter during hypoxia (7). Here, we demonstrate that endogenous Hif-1α binds to HRE1 and HRE4 of the PD-L1 promoter in LPS-stimulated primary macrophages.

Moreover, endogenous PKM2–chromatin complexes isolated from LPS-stimulated primary BMDM cells show a clear increase in binding to the HRE1 as well as HRE4 promoter regions of PD-L1, as well as a significant upregulated association with the VEGF Hif-1α-binding site in the VEGF promoter site compared to control (Figure 2B, right).

To determine the simultaneous binding of Hif-1α and PKM2 to the same region of the PD-L1 promoter, a sequential ChIP or re-ChIP was performed. We observed an increased concurrent binding of Hif-1α and PKM2 to two HRE elements in the PD-L1 promoter (Figure 2C). Interestingly, TEPP-46 not only prevents PKM2 from binding to the PD-L1 promoter but conversely Hif-1α also requires mono/dimeric PKM2 to successfully interact with the promoter sites (Figure S2 in Supplementary Material).

### PKM2 Regulates Expression of PD-L1 in Immune as well as Murine Tumor Cells

**Figure 3:**
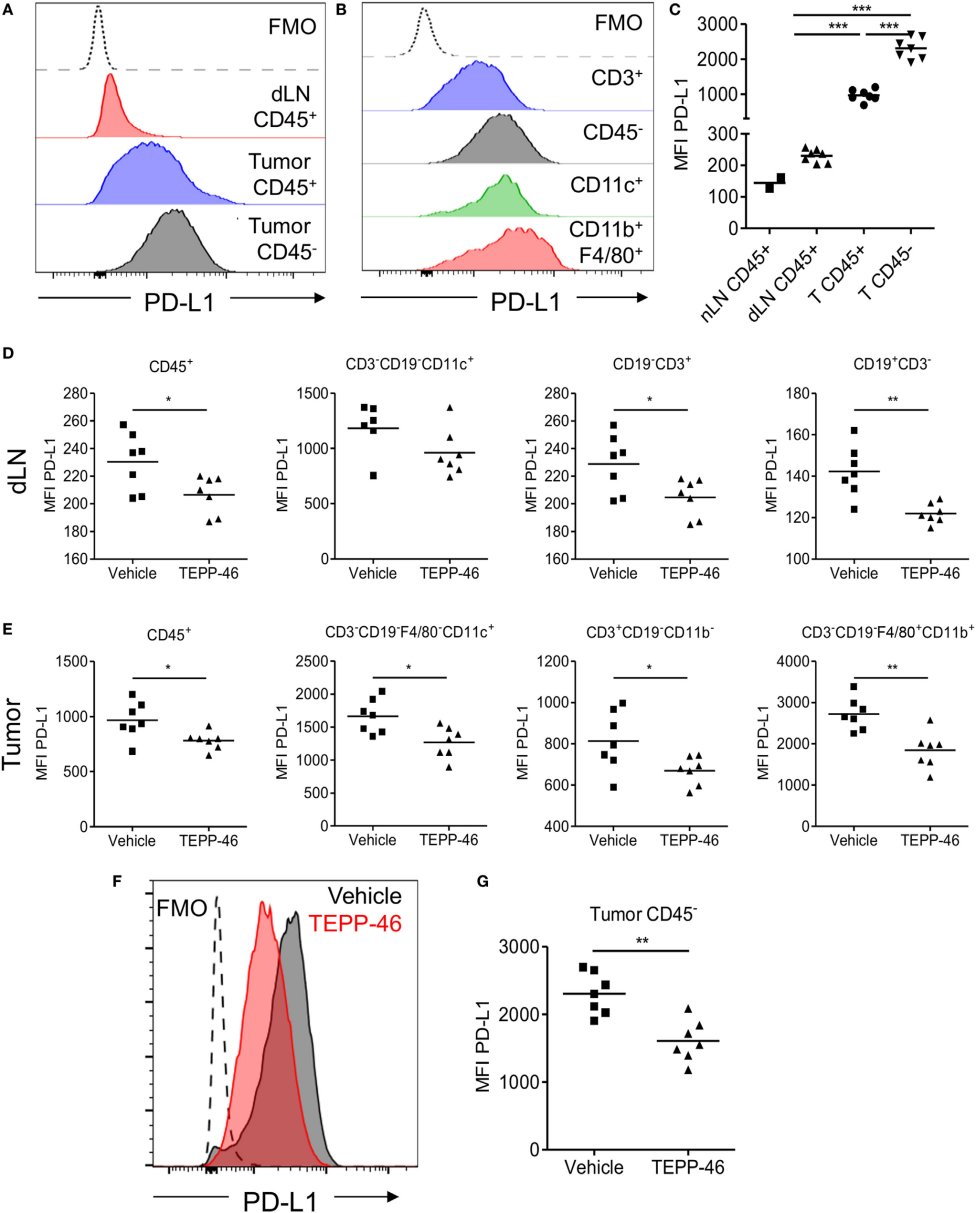

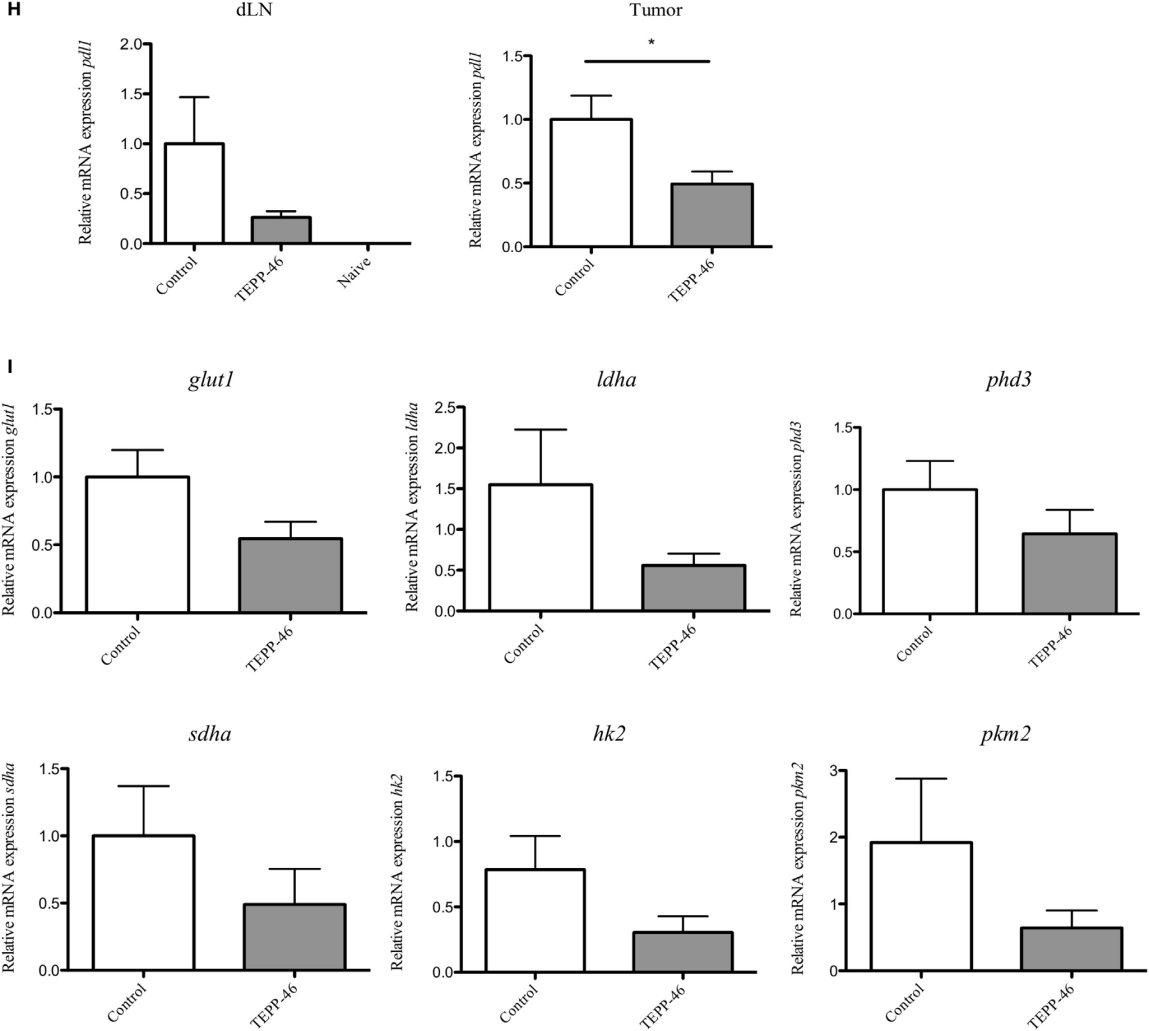
TEPP-46 reduces PD-L1 expression on tumor and immune cells *in vivo* in the CT26 colon carcinoma animal model. Single cell suspensions from CT26 tumors or dLN isolated on day 15 post-tumor induction were stained for flow cytometry **(A–C)**. Representative FACS histograms of PD-L1 cell surface expression on CD45^−^ or CD45^+^ cells from tumor dLN or tumors **(A)**. Representative FACS histograms of PD-L1 cell surface expression on different CT26 infiltrating immune cell populations **(B)**. **(C)** PD-L1 MFI for CD45^−^ or CD45^+^ cells from naïve LN, dLN, or tumors (T). Mice were inoculated with CT26 tumors and injected i.p. with vehicle or TEPP-46 every 2–3 days, beginning on day −1 pre-tumor induction **(D–I)**. Tumors and dLNs were isolated on day 15 post-tumor induction. MFI of PD-L1 cell surface expression on different immune populations in dLN **(D)** or tumors **(E)**. Representative histogram of PD-L1 expression on CD45^−^ cell from vehicle or TEPP-46-treated tumors **(F)**. PD-L1 MFI for CD45^−^ cells from tumors **(G)**. Total *pdl1* RNA expression in cells isolated from dLN [**(H)**, left] or tumor [**(H)**, right]. Statistics are performed as one-way ANOVA with Bonferroni post-test **(C)** and two-tailed unpaired *t*-test **(D,E,G,H)**; *P < 0.05, **P < 0.01, and ***P < 0.001. **(I)** Expression of proglycolytic and Hif-1α-dependent genes (*glut1, ldha*, and *phd3* top panels, *sdha, hk2*, and *pkm2*, bottom panels) in tumor biopsies isolated on day 15 post-tumor induction.

PD-1 and its ligand PD-L1 play an important role in the subversion of antitumor immune responses. The murine CT26 colon carcinoma model was used to study the role of PKM2 in the regulation of PD-L1 expression in an *in vivo* setting. Flow cytometric analysis of CT26 tumor-bearing mice revealed that immune cells (CD45^+^) expressed increased levels of PD-L1 in dLN compared with naïve LN (Figure 3C). The expression level of PD-L1 was more dramatically increased on tumor-infiltrating immune cells compared to LN cells (Figures 3A,C), suggesting that the tumor microenvironment promotes PD-L1 expression. Tumor and stromal cells (CD45^−^) expressed the highest levels of PD-L1 compared with immune cells (Figures 3A,C). Of the tumor-infiltrating immune populations, CD3^+^ T cells expressed the lowest levels of PD-L1, while CD11c^+^ DCs or CD11b^+^F4/80^+^ monocytes/macrophages expressed the highest levels of PD-L1 (Figure 3B). To test whether PD-L1 expression in the CT26 tumor model was regulated by PKM2, mice were injected with TEPP-46 every 2–3 days. TEPP-46 effectively reduced the expression of PD-L1 in CD45^+^ leukocytes, CD11c^+^ DCs, CD3^+^ T cells, or CD19^+^ B cells in the dLN (Figure 3D). Moreover, of the cells occupying the tumor microenvironment, TEPP-46 significantly downregulated PD-L1 expression on leukocytes in general (CD45^+^) and specifically on CD11c^+^ DCs, CD3^+^ T cells, or F4/80^+^CD11b^+^ monocytes/macrophages (Figure 3E). Interestingly, TEPP-46 also downregulated PD-L1 expression on CD45^−^ cells, which comprise tumor and stromal cells (Figures 3F,G). In addition, overall PD-L1 mRNA expression levels measured by RT-PCR in dLN and tumor samples were reduced (Figure 3H).

mRNA isolated from tumors from mice treated with TEPP-46 also displayed a blunted expression level of a range of proglycolytic genes in the tumor microenvironment. These include the glucose transporter GLUT-1, lactate dehydrogenase A (LDHA), succinate dehydrogenase complex A (SDHA), hexakinase-2, PKM2, and PHD3 (Figure 3I), suggesting that inhibition of PKM2 may also restrain the highly glycolytic metabolic profile displayed by many tumors.

## Discussion

There are extensive reports pointing to a role for PKM2 in cell proliferation and tumor progression, where PKM2 is essential for aerobic glycolysis, the dominant metabolic pathway utilized by cancer cells. However, recent studies using PKM2^fl^ conditional allele knockout mice suggest that the precise role for PKM2 in cancer growth is controversial (19). Work is currently underway to characterize the role of PKM2 in immune cells. Notably, PKM2 is imperative to macrophage function, enabling the critical metabolic shift from oxidative phosphorylation to aerobic glycolysis in LPS-activated macrophages (20), promoting HMGB1 release during sepsis (21), as well as contributing to pathogenesis of hyper-inflammatory macrophages in coronary artery disease (22).

Pyruvate kinase isoform M2 promotes transactivation of Hif-1α responsive genes by participating in the assembly of a nuclear multiprotein complex consisting of PKM2, PHD3, p300, and Hif-1α (23) stimulating upregulation of proglycolytic genes such as *glut1, ldha*, and *pdk1* in both cancer cells and in primary macrophages (11, 20).

Induction of PD-L1 in response to LPS on macrophages and DCs as observed here suggests a role for PD-L1 in fine-tuning responses to infection. Furthermore, this induction of PD-L1 requires expression of dimeric PKM2, since a conformational change of PKM2 into a tetramer aided by TEPP-46, or silencing expression of PKM2 mRNA prevents LPS-induced PD-L1 expression. To decipher the mechanism by which PKM2 regulates expression of PD-L1, we hypothesized that PKM2 would bind to the same regions of the PD-L1 promoter as reported for Hif-1α. Indeed, we observed a strong interaction of PKM2, as well as Hif-1α, with two HRE-binding sites in the PD-L1 promoter in LPS-treated macrophages. In addition, we here demonstrate simultaneous and reciprocally codependent binding of Hif-1α and PKM2 to two HRE sites in the promoter of PD-L1.

Our findings demonstrate that PD-L1 is widely expressed *in vivo* in the context of a growing CT26 tumor and is upregulated on immune and tumor cells in the tumor microenvironment. Induction of PKM2 tetramerization, which inhibits PKM-2 targeted gene expression, reduced the expression of PD-L1, suggesting that the hypoxic tumor microenvironment promotes widespread PD-L1 expression through PKM2. TEPP-46 treatment did not significantly alter tumor growth in this setting (data not shown); however, in line with published observations that anti-PD1 treatment alone does not reduce tumor growth in this model (24) this may not have been expected. Importantly, these findings give insight into a novel mechanism regulating PD-L1 expression in a range of cells in the tumor microenvironment. These findings are consistent with reports that hypoxia-induced HIF-1α promotes PD-L1 expression on macrophages, DCs, and murine and human tumor cells (7). Moreover, blockade of HIF-1α enhanced the efficacy of a DC vaccine in a murine breast cancer model, an effect that could be due to reduction of PD-L1 by blocking HIF-1α (25).

Our findings here identify PKM2 as a novel regulator of LPS- as well as tumor-induced PD-L1 expression on macrophages and DCs as well as tumor cells, providing new insight into the expressional control of an important target in cancer immunotherapy.

## Ethics Statement

All mice used were maintained under specific pathogen-free conditions in line with Irish and European Union regulations. All experiments were carried out with prior ethical approval from Trinity College Dublin Animal Research Ethics Committee.

## Author Contributions

EP-M designed and performed experiments, analyzed data, and wrote the manuscript. LD designed and performed experiments, analyzed data, and cowrote the manuscript. ZZ, DM, and AM performed experiments. KM conceived ideas. LO conceived ideas, oversaw the project, and cowrote the manuscript.

## Conflict of Interest Statement

The authors declare that the research was conducted in the absence of any commercial or financial relationships that could be construed as a potential conflict of interest.

## Supplementary Material

Figure S1 Gating strategy for tumor-infiltrating immune cell populations.

Figure S2 Chromatin immunoprecipitation-PCR on lysates from TEPP-46 (pretreatment using 50 μM, 60 min) and LPS-treated BMDMs (100 ng/ml, 24 h) using a HIF-1α-specific antibody and primers specific for two promoter regions of PD-L1 (HRE1 and 4) showing binding of endogenous Hif-1α to the PD-L1 promoter.

Table S1 List of antibodies used for flow cytometry analysis of in vivo tissues.

Table S2 List of SYBR probes used for RNA analysis.

Table S3 Sequences of oligonucleotides used in OPD assay.
