# Supplementary material for: Pyruvate Kinase M2 Is Required for the Expression of the Immune Checkpoint PD-L1 in Immune Cells and Tumors
Source: Front Immunol. 2017 Oct 13;8:1300. doi: 10.3389/fimmu.2017.01300 (PMC5646285; doi:10.3389/fimmu.2017.01300)
Supplement: Figure S1 — Gating strategy for tumor-infiltrating immune cell populations. [file Presentation_1.PPTX]

## Slide 1
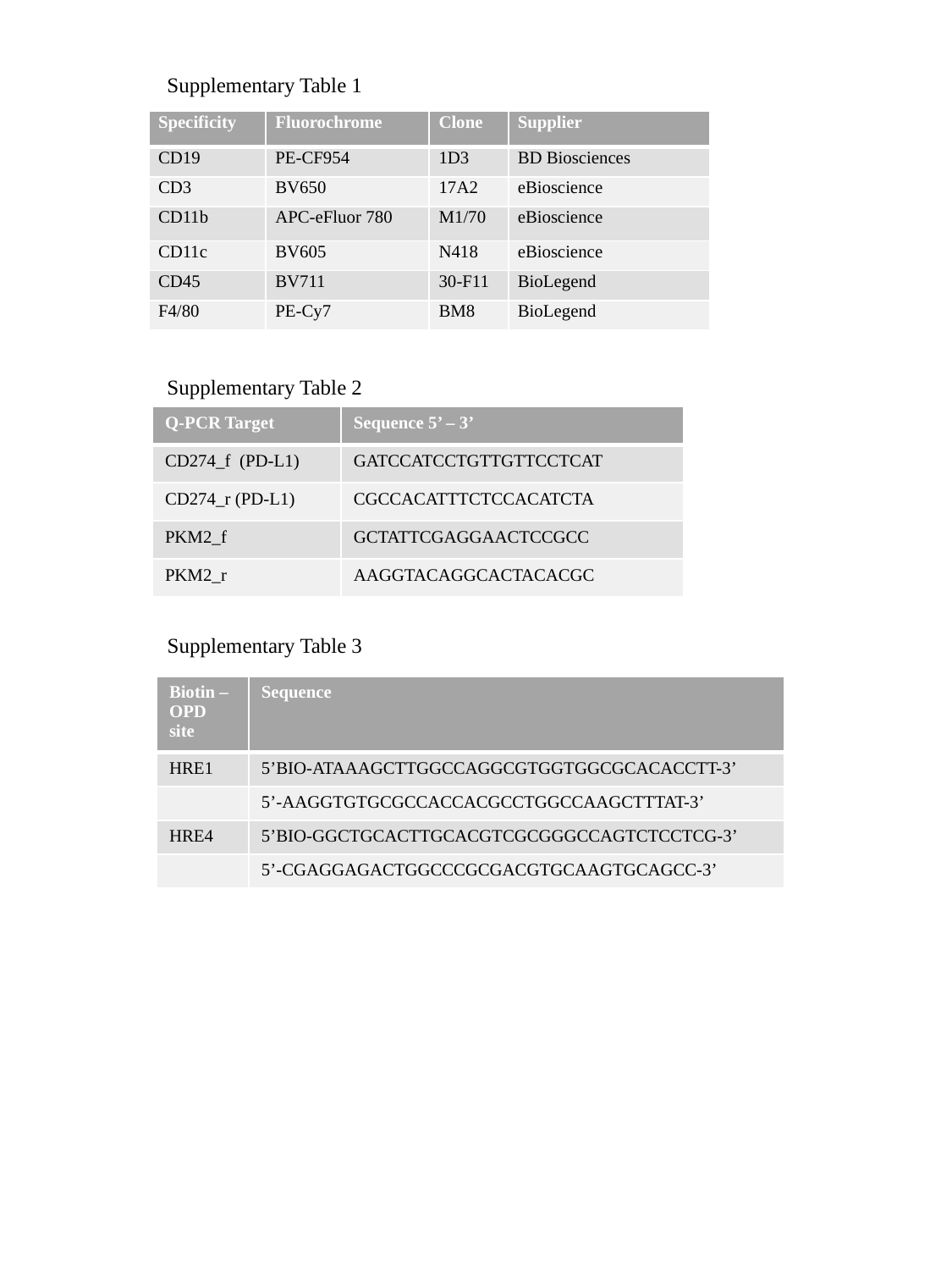

Supplementary Table 1
| Specificity | Fluorochrome | Clone | Supplier |
| --- | --- | --- | --- |
| CD19 | PE-CF954 | 1D3 | BD Biosciences |
| CD3 | BV650 | 17A2 | eBioscience |
| CD11b | APC-eFluor 780 | M1/70 | eBioscience |
| CD11c | BV605 | N418 | eBioscience |
| CD45 | BV711 | 30-F11 | BioLegend |
| F4/80 | PE-Cy7 | BM8 | BioLegend |
Supplementary Table 2
| Q-PCR Target | Sequence 5’ – 3’ |
| --- | --- |
| CD274\_f (PD-L1) | GATCCATCCTGTTGTTCCTCAT |
| CD274\_r (PD-L1) | CGCCACATTTCTCCACATCTA |
| PKM2\_f | GCTATTCGAGGAACTCCGCC |
| PKM2\_r | AAGGTACAGGCACTACACGC |
Supplementary Table 3
| Biotin – OPD site | Sequence |
| --- | --- |
| HRE1 | 5’BIO-ATAAAGCTTGGCCAGGCGTGGTGGCGCACACCTT-3’ |
| | 5’-AAGGTGTGCGCCACCACGCCTGGCCAAGCTTTAT-3’ |
| HRE4 | 5’BIO-GGCTGCACTTGcacgtcgcgGGCCAGTCTCCTCG-3’ |
| | 5’-CGAGGAGACTGGCCCGCGACGTGCAAGTGCAGCC-3’ |

## Slide 2
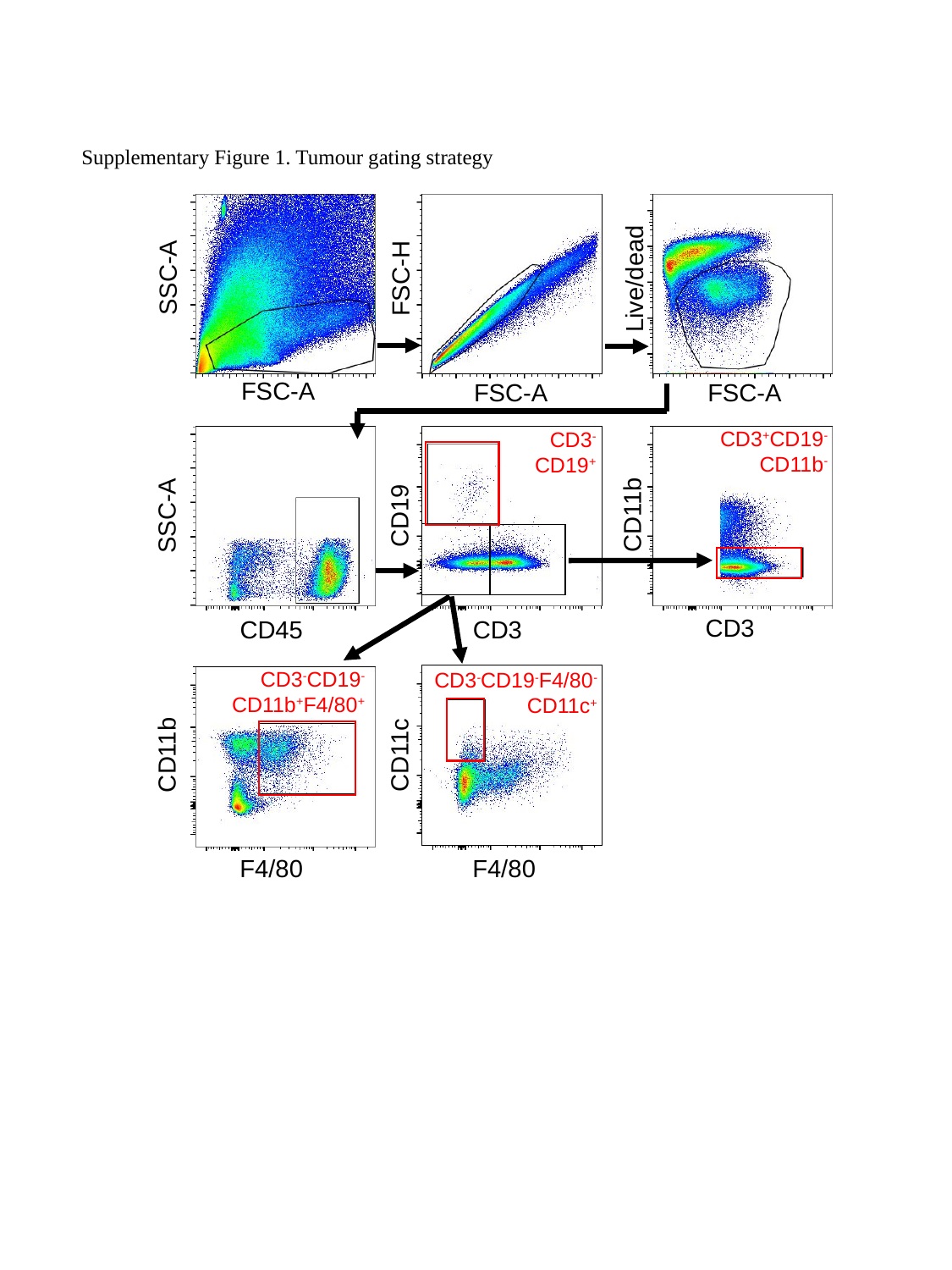

Supplementary Figure 1. Tumour gating strategy
SSC-A
FSC-H
Live/dead
FSC-A
FSC-A
FSC-A
CD3+CD19-
CD11b-
CD3-
CD19+
CD11b
SSC-A
CD19
CD3
CD45
CD3
CD3-CD19-
CD11b+F4/80+
CD3-CD19-F4/80-
CD11c+
CD11b
CD11c
F4/80
F4/80

## Slide 3
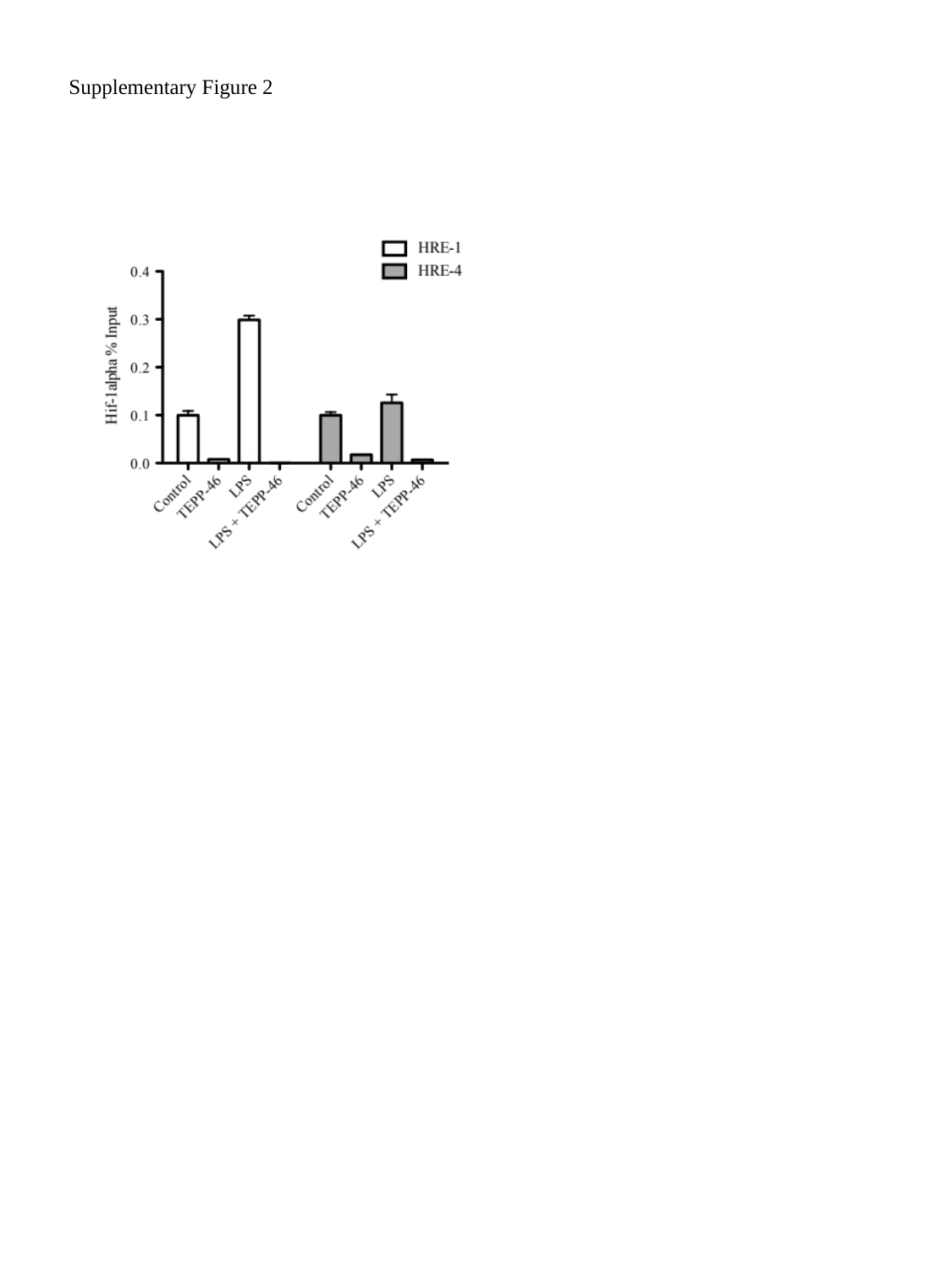

Supplementary Figure 2
